# Frequency distribution of *IL*-*17A* G197A (rs2275913) and *IL*-*17F* A7488G (rs763780) polymorphisms among healthy Sudanese population

**DOI:** 10.1186/s13104-020-05165-4

**Published:** 2020-07-02

**Authors:** Nouh S. Mohamed, Emmanuel E. Siddig, Abdallah E. Ahmed, Musab M. A. Albsheer, Hanadi Abdelbagi, Eman T. Ali, Anadel A. Alsubki, Sabah A. Abdalaziz, Madinna Mustafa, Mohamed S. Muneer, Hussam A. Osman, Maha M. Osman, Mohamed S. Ali, Ali M. M. Edris, Ayman Ahmed, Rihab A. Omer

**Affiliations:** 1 Department of Parasitology and Medical Entomology, Faculty of Medical Laboratory Sciences, Nile University, Khartoum, Sudan; 2 Alfarrabi College for Science and Technology, Khartoum, Sudan; 3 Department of Parasitology and Medical Entomology, Faculty of Medicine, Sinnar University, Sinnar, Sudan; 4 School of Medicine, Nile University, Khartoum, Sudan; 5 Mycetoma Research Center, University of Khartoum, Khartoum, Sudan; 6 Department of Biotechnology, School of Pharmacy, Ahfad University for Women, Omdurman, Sudan; 7 Department of Histopathology and Cytology, Faculty of Medical Laboratory Sciences, University of Khartoum, Khartoum, Sudan; 8 Department of Neurology, Mayo Clinic, Jacksonville, FL USA; 9 Department of Radiology, Mayo Clinic, Jacksonville, FL USA; 10 Department of Internal Medicine, Faculty of Medicine, University of Khartoum, Khartoum, Sudan; 11 Faculty of Medicine, Neelain University, Khartoum, Sudan; 12 Department of Histopathology and Cytology, Faculty of Applied Medical Sciences, University of Bisha, Bisha, Kingdom of Saudi Arabia; 13 Institute of Endemic Diseases, University of Khartoum, Khartoum, Sudan; 14 Department of Molecular Biology, Institute of Parasitology, University of Leipzig, Leipzig, Germany

**Keywords:** Interleukin 17A, Interleukin 17F, Polymorphism, Healthy population, Sudan

## Abstract

**Objectives:**

*IL*-*17A G197A* and *IL*-*17F A7488G* polymorphisms has been identified to be associated with the susceptibility to many diseases. This study aimed to investigate the frequency distribution of IL-17A G197A and IL-17F A7488G polymorphisms among healthy Sudanese population. A descriptive cross-sectional hospital-based molecular study conducted in different sites throughout Sudan. Two ml blood samples were collected from 717 healthy participants. Demographic data and the medical history of the participants were collected.

**Results:**

Of the 717 participants, 355 (49.5%) were males and 362 (50.5%) were females, their mean age was 30.2 ± 17.2 and 32.2 ± 16.5, respectively. For *IL*-*17A*, the most frequent genotype detected among males and females was *IL*-*17A* heterozygote allele (AG); 215 (60.6%) and 194 (53.6%), respectively. Whereas, for *IL*-*17F*, the most frequent allele among males and females was the homozygote allele (AA); 298 (83.9%) for males and 322 (89.0%) for females. HWE for genotype distributions of *IL*-*17A* was showing statistical insignificance for *IL*-*17A* among males and females, P value 0.614. While HWE for *IL*-*17F* reached the equilibrium level, P value 0.048. The most frequent age group was those aged between 21 to 40 years; 281 (39.2%). Arab constituted the major ethnicity of the study participants; 418 (58.3%), P value 0.034.

## Introduction

T helper 17 (Th17) cells, is one of the CD4 T helper cells lineages that been defined as a unique effector subset of cells [1], in particular through the production of Interleukins (ILs) mainly *IL*-*17A* and *IL*-*17F* [1, 2]. The *IL*-*17* family of cytokines contains other 4 members including; *IL*-*17B, IL*-*17C, IL*-*17D*, and *IL*-*17E* [2]. Both *IL*-*17A* and *IL*-*17F* are considered as inflammation-related genes [3]. Although little is known about most of the *IL*-*17* family members, *IL*-*17F* was discovered to share the strongest homology to *IL*-*17A*. The previous report on *IL*-*17A* and *IL*-*17F* in inducing the expression of other various adhesion molecules, cytokines, and chemokines was reported [4]. Previously, polymorphisms of *IL*-*17A G197A* (rs2275913) and *IL*-*17F A7488G* (rs763780) were found to be associated with the increased susceptibility to rheumatoid arthritis and ulcerative colitis, respectively [5, 6]. Also, *IL*-*17A* and *IL*-*17F* were investigated in gastric cancer risks and the association of each single nucleotide polymorphism (SNP) with subtypes of gastric cancer according to its clinicopathological features and their roles in prognosis [7]. *IL*-*17A* and *IL*-*17F* have also been associated with the pathogenesis of a growing list of autoimmune and inflammatory diseases, such as inflammatory bowel diseases and psoriasis [8, 9]. Several studies have found excess expression of *IL*-*17A* in various tumor tissues, including prostate cancer, colorectal cancer, breast cancer, and gastric cancer [10–13]. Moreover, increasing evidence suggested the role of *IL*-*17A* in *Helicobacter pylori*-related gastric diseases [14, 15].

In Sudan, no study has ever investigated the frequency distribution of *IL*-*17A G197A* (rs2275913) and *IL*-*17F A7488G* (rs763780) polymorphisms among the Sudanese population. In a previous study conducted by Wu et al. [7] provided the first evidence that the *IL*-*17F A7488G* coding variant increases gastric cancer risks in a low-risk Chinese population, and revealed its association with subtypes of clinicopathologic features of the gastric cancer patients. Studies are needed to investigate the distribution of *IL*-*17A G197A* (rs2275913) and *IL*-*17F A7488G* (rs763780) polymorphisms. Also, the result of the known population structure of this gene has implications for understanding the epidemiology not only of cancer, but also the increased susceptibility towards gastric inflammations in Sudan, and the potentials for more effective treatment therapy. In this study, we aimed to determine the frequency of *IL*-*17A G197A* (rs2275913) and *IL*-*17F A7488G* (rs763780) polymorphisms among a healthy Sudanese population.

## Main text

### Materials and methods

#### Study design, study sites, samples and data collection

This is a descriptive cross-sectional hospital-based molecular study conducted in different sites throughout Sudan including; Khartoum and Madani (central region); New Halfa, Port Sudan, and Gedaref (eastern region); River Nile (North region); and Ad Damazin and Kosti (southern region). Two ml blood samples were collected from 717 healthy participants recruited at the health facilities of each site. Blood samples were preserved in sodium citrate blood containers. Demographic data and the medical history of the participants were collected. Participation in this study was fully voluntary, and only individuals who expressed interest willingly to participate in this study by signing a written informed consent form were included in the study. Pregnant women, children aged less than 1 year, participants with a history of ulcerative colitis and rheumatoid arthritis, beside immune-compromised patients were excluded from the study to reduce hemoglobin loss in case of pregnant women and infants and to avoid bias in the results of SNPs frequency distribution in case of those with history of ulcerative colitis and rheumatoid arthritis.

### PCR-RFLP for *IL*-*17F A7488G* and *IL*-*17A G197A* genotyping

The genomic DNA was extracted from blood samples using QIAamp DNA blood Mini Kit (Qiagen Inc., Germany). DNA was re-suspended in 200 μl of 1X TE-buffer and stored at − 20 °C until molecular investigations. *IL*-*17A G197A* and *IL*-*17F A7488G* genotyping was performed by polymerase chain reaction-restriction fragment length polymorphism (PCR–RFLP). Primers used for *IL*-*17A G197A* and *IL*-*17F A7488G* were as follows: sense 5-AACAAGTAAGAATGAAAAGAGGACATGGT-3 and anti-sense 5-CCCCCAATGAGGTCATAGAAGAATC-3 for *IL*-*17A*; sense 5-ACCAAGGCTGCTCTGTTTCT-3 and anti-sense 5-GGTAAGGAGTGGCATTTCTA-3 for *IL*-*17F* as described previously [7]. The PCR amplification was performed in a total volume of 25 µl mixture containing using single tube PCR i-Taq premix (iNtRON Biotechnology, Korea), mixed with 1 µl genomic DNA, and 1 µM of each primer and incubated in MJ research thermocycler (USA) using the previously described amplification condition [7]. PCR products were digested overnight at 37 ℃ with *XagI* and *NIaIII* (New England BioLabs, England) to determine the genotypes of *IL*-*17A G197A* and *IL*-*17F A7488G*, respectively. Digested amplicons were separated using 3% agarose gel electrophoresis. To confirm the genotyping results, randomly selected PCR products were sequenced using Sanger deoxy ribonucleic acid sequencing method using ABI 3730 sequencing system provided by BGI company (BGI, China).

### Statistical analysis

Data were analyzed using the Statistical Package for the Social Sciences (SPSS v20). Hardy-Weinberg equilibrium (HWE) was performed using Pearson’s χ^2^ test. Differences in allele frequency were analyzed using Fisher’s exact test, a P value < 0.05 was considered significant. The sequences of *IL*-*17A* and *IL*-*17F* products were analyzed using BioEdit v7 software for the confirmation of sequences polymorphisms.

## Results

### PCR-RFLP and sequencing results

**Fig. 1:**
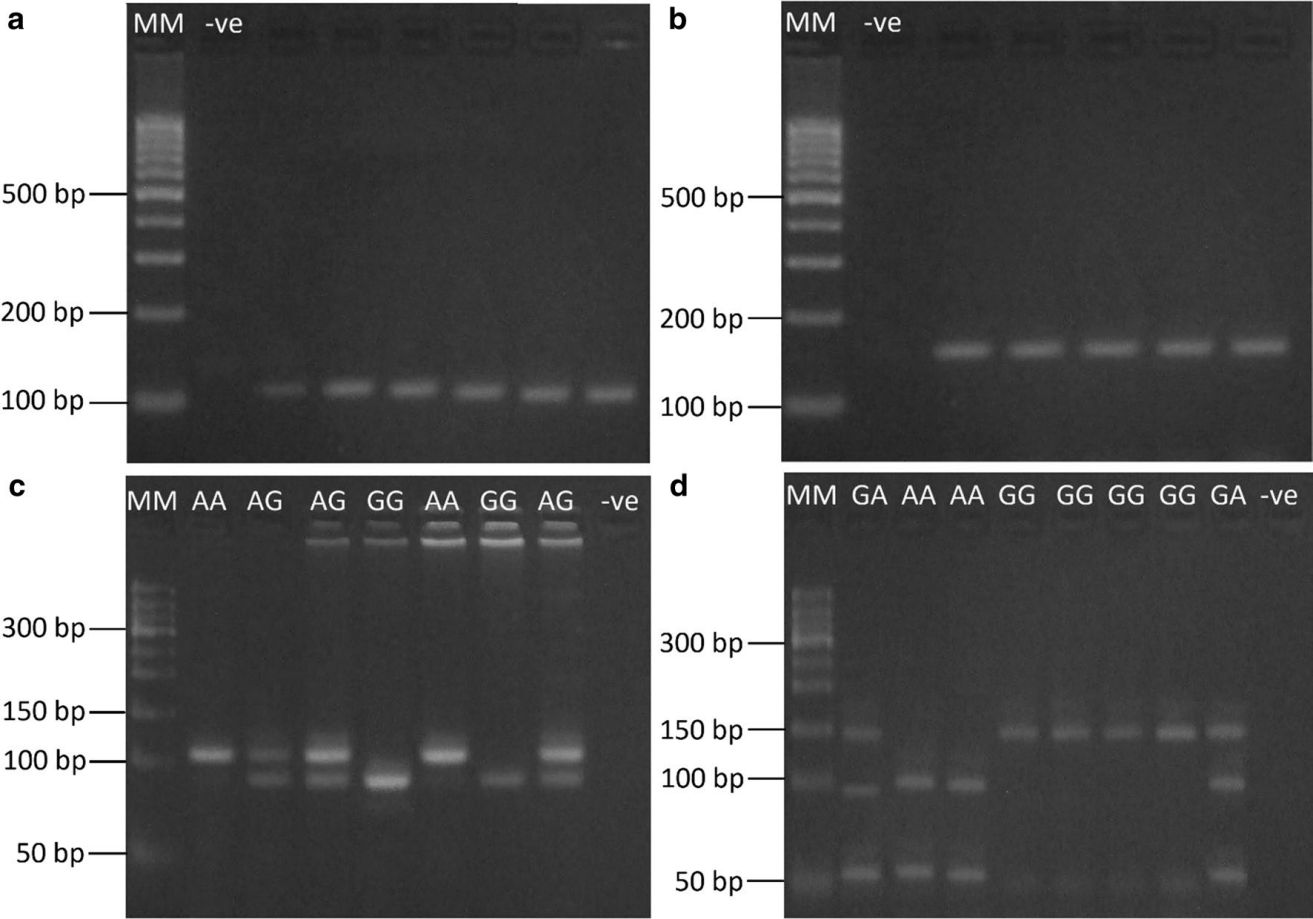
PCR-RFLP results of *IL*-*17A* and *IL*-*17F* genotyping

Amplified PCR products of each of *IL*-*17A G197A* and *IL*-*17F A7488G* bands sizes were 102 and 143 base pairs (bp), respectively. The results of enzyme digestion using *XagI* and *NIaIII* produced several fragments grouped into 3 types of fragments (Fig. 1).

**Fig. 2:**
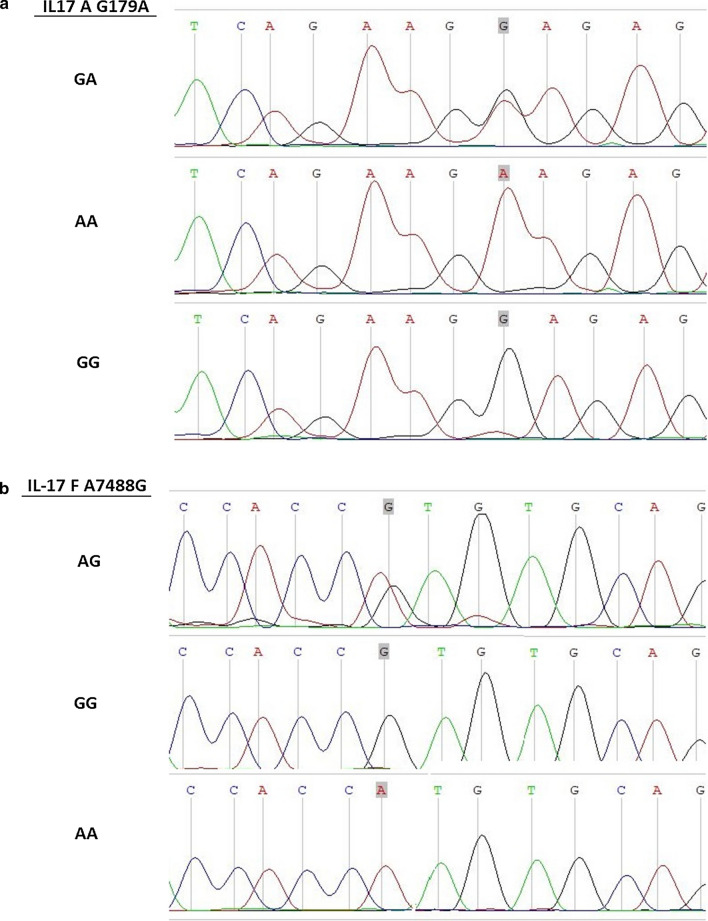
Sequencing results of the amplified products for *IL*-*17A* and *IL*-*17F* polymorphisms

The selected samples were accurately confirming the cutting sites of each enzyme, and showing the correct SNP (Fig. 2).

### Frequency of *IL*-*17A G197A* and *IL*-*17F A7488G* genotypes

In this study a total of 717 healthy participants were included. 355 (49.5%) were males and 362 (50.5%) were females, their mean age was 30.2 ± 17.2 and 32.2 ± 16.5, respectively.

For *IL*-*17A*, the most frequent genotype detected among males and females was *IL*-*17A* heterozygote allele (AG); 215 (60.6%) and 194 (53.6%), respectively. Whereas, for *IL*-*17F*, the most frequent allele among males and females was the homozygote allele (AA); 298 (83.9%) for males and 322 (89.0%) for females. No statistical significance association for frequency distribution of the different *IL*-*17A* and *IL*-*17F* genotypes based on gender, P values were 0.113 and 0.136, respectively. HWE for genotype distributions of *IL*-*17A* was showing statistical insignificance for *IL*-*17A* among males and females, HWE (Fisher exact test = 0.614). While HWE for *IL*-*17F* reached the equilibrium level, HWE (Fisher exact test = 0.048).

Based on age groups, the most frequent age group was those aged between 21 to 40 years; 281 (39.2%), followed by participants aged between 1 to 21 years; 188 (26.2%). Concerning the ethnicity, Arab constituted the major ethnicity of the study participants; 418 (58.3%). Frequency distribution across the different ethnic groups was statistically significant for *IL*-*17A* genotypes, P value 0.034 (Table 1).

**Table 1:** Frequency distribution of *IL*-*17A* and *IL*-*17F* genotypes among participants gender, age groups and participants ethnicity

	IL-17 A genotypes no. (%)				IL-17 F genotypes no. (%)				Total
	AA	AG	GG	P*	AA	GA	GG	P*	
Gender									
Male	127 (35.8)	215 (60.6)	13 (3.7)	0.113	298 (83.9)	42 (11.8)	15 (4.2)	0.136	355 (49.5)
Female	147 (40.6)	194 (53.6)	21 (5.8)		322 (89.0)	28 (7.7)	12 (3.3)		362 (50.5)
Age group									
1–20 years	71 (37.8)	108 (57.4)	9 (4.8)	0.977	166 (88.3)	15 (8.0)	7 (3.7)	0.231	188 (26.2)
21– 40 years	110 (39.1)	160 (56.9)	11 (3.9)		231 (82.2)	37 (13.2)	13 (4.6)		281 (39.2)
41–60 years	54 (40.0)	74 (54.8)	7 (5.2)		118 (87.4)	13 (9.6)	4 (3.0)		135 (18.8)
61–80 years	9 (30.0)	19 (63.3)	2 (6.7)		29 (96.7)	1 (3.3)	0 (0.0)		30 (4.2)
81–100 years	30 (36.1)	48 (57.8)	5 (6.0)		76 (91.6)	4 (4.8)	3 (3.6)		83 (11.6)
Ethnicity									
Arab	142 (34.0)	257 (61.5)	19 (4.5)	0.034	363 (86.8)	40 (9.6)	15 (3.6)	0.992	418 (58.3)
Beja	8 (30.8)	16 (61.5)	2 (7.7)		23 (88.5)	2 (7.7)	1 (3.8)		26 (3.6)
Fallata	8 (40.0)	11 (55.0)	1 (5.0)		17 (85.0)	3 (15.0)	0 (0.0)		20 (2.8)
Fur	30 (50.8)	26 (44.1)	3 (5.1)		50 (84.7)	6 (10.2)	3 (5.1)		59 (8.2)
Nuba	34 (35.1)	59 (60.8)	4 (4.1)		82 (84.5)	11 (11.3)	4 (4.1)		97 (13.5)
Nubian	52 (53.6)	40 (41.2)	5 (5.2)		85 (87.6)	8 (8.2)	4 (4.1)		97 (13.5)

*P, P value

The subgroup analysis of IL-17A and IL-17F genotypes distribution across the different ethic groups revealed a statistically significant difference of IL-17A genotypes among Arabs compared to the different ethic groups (See Additional file 1). Whereas, no statistically significant difference obtained for IL-17F genotypes distribution across the different Sudanese ethnic groups (Additional file 2).

## Discussion

The polymorphisms of *IL*-*17A G197A* (rs2275913) and *IL*-*17F A7488G* (rs763780) has been associated with susceptibility to various types of proinflammatory diseases and gastric cancer [5–7]. Additionally, knowing the population structure of this gene was noted to be uniquely beneficial in means of treatment and understanding diseases prognosis [16]. In this study, we aimed to determine the frequency of *IL*-*17A* and *IL*-*17F* polymorphisms among healthy Sudanese populations. The results obtained in this study, revealed that the distribution of *IL*-*17A* and *IL*-*17F* was statistically insignificant among males and females. This agreeing with previously conducted studies [17, 18]. Although, the study participants were selectively healthy individuals this could provide a hint towards the chance of difference occurrence when including unhealthy individuals diagnosed with gastric cancer, breast cancer or rheumatoid arthritis [7, 17, 19, 20].

Interestingly, this result also supports the fact that this gene could be significantly linked with susceptibly to certain diseases such as *H. pylori* infection [21]. Since *H. pylori* infections reported in Sudan are quietly increasing [22–25], and here, the high frequency of *IL*-*17A* and *IL*-*17F* genotypes can increase the susceptibility towards *H. pylori* infection. However, this assumption needs further investigations.

Regarding, the distribution among the different age groups obtained in this study, although, no association was found, *IL*-*17A* polymorphism has been associated with early TNM staging and poorly differentiated gastric cancers with aging [7]. Moreover, this result showed the degree of population structure for this gene which will help in cancer onset prediction especially among elderly. This was also seen by the small number of elderlies been included hence most elders were diagnosed previously with rheumatoid arthritis and gastric colitis and been excluded from the study. This was in line with the previous hypotheses that *IL*-*17A* polymorphism may influence the development and progression of gastric carcinogenesis [7]. Also, the *IL*-*17F 7488GA* genotype that reported to increase gastric cancer risk from the age of 40 years [7].

The significant association between *IL*-*17A* and participants ethnicity could be attributed to several factors, remarkably, based on 2009–2010 cancer prevalence in Sudan, 37.8% of the total cancer patients were diagnosed with breast cancer [26]. Although, less is known about patient’s ethnicity, nevertheless based on Mahmoud et al. (unpublished data), the rate of breast and gastric cancer incidence in Sudan since 2010 to 2015 were approximately 20% and 5% respectively. Among both cancer groups, 60% and 70% were noted to be of Arab ethnicity (unpublished data). This is suggesting that *IL*-*17A* polymorphism is taking role in cancer susceptibility among different Sudanese ethnic groups. This was well discussed previously by Li et al. [21], indicating the role of ethnicity in gastric cancer susceptibly, where found that *IL*-*17A* increases gastric cancer susceptibility among Japanese population but not with the Chinese population.

This study highlights the need for further investigations towards addressing *IL*-*17A* and *IL*-*17F* polymorphisms among Sudanese population diagnosed with different types of cancers, in order to further understand the role of this SNP polymorphism in cancer susceptibility and further cancer incidence. However, such prediction could also be ambiguous and misleading since cancer susceptibility may not be linked to a single nucleotide polymorphism at a single gene [27, 28].

## Conclusion

This study provides the first data on the Sudanese population structure on *IL*-*17A* and *IL*-*17F* gene polymorphisms. This might be of help to identify the association of these polymorphisms with different patients’ groups and could benefit in the understanding of those genes’ involvement in caner or other types of diseases susceptibility.

## Limitations

- This study focused on healthy Sudanese participants, limiting the natural variation within the population structure, which might be biasly selected against the *IL*-*17A* and *IL*-*17F* polymorphisms and resulted in the underestimation of their prevalence. Therefore, the need for more diverse study population including unhealthy population is extremely important to further understand the role of these SNPs in different diseases’ susceptibility.

## Supplementary information

**Additional file 1: Table S1.** Subgroup analysis of *IL-17A* genotypes distribution across the different Sudanese ethnic groups.

**Additional file 2: Table S2.** Subgroup analysis of *IL-17F* genotypes distribution across the different Sudanese ethnic groups. M±Std: Mean Difference ± Standard Error. 95% CI [L-U]: 95% Confidence Interval [ Lower bound -Upper bound].

## Data Availability

The datasets used and/or analyzed during the current study are available from the corresponding author on reasonable request.

## Abbreviations

HWE: Hardy–Weinberg Equilibrium
ILs: Interleukins
PCR-RFLP: Polymerase Chain Reaction-Restriction Fragment Length Polymorphism
SNP: Single Nucleotide Polymorphism
SPSS: Statistical Package for Social Sciences
Th17: T helper 17
