# Supplementary material for: Frequency distribution of IL-17A G197A (rs2275913) and IL-17F A7488G (rs763780) polymorphisms among healthy Sudanese population
Source: BMC Res Notes. 2020 Jul 2;13:317. doi: 10.1186/s13104-020-05165-4 (PMC7330939; doi:10.1186/s13104-020-05165-4)
Supplement: Supplementary file 1 — Additional file 1: Table S1. Subgroup analysis of IL-17A genotypes distribution across the different Sudanese ethnic groups. [file 13104_2020_5165_MOESM1_ESM.docx]

|  | **Arab** | **Beja** | **Fallata** | **Fur** | **Nuba** |
| --- | --- | --- | --- | --- | --- |
| **Beja** | | | | | |
| M±Std | -0.063±0.113 | - | - | - | - |
| 95% CI [L-U] | -0.29 – 0.16 | - | - | - | - |
| P value | **0.006** | - | - | - | - |
| **Fallata** | | | | | |
| M±Std | 0.056±0.128 | 0.119±0.167 | - | - | - |
| 95% CI [L-U] | -0.20 – 0.31 | -0.21 – 0.45 | - | - | - |
| P value | **0.041** | 0.475 | - | - | - |
| **Fur** | | | | | |
| M±Std | 0.163±0.078 | 0.227±0.132 | 0.108±0.145 | - | - |
| 95% CI [L-U] | 0.01 – 0.32 | -0.03 – 0.49 | -0.18 – 0.39 | - | - |
| P value | **0.037** | 0.086 | 0.458 | - | - |
| **Nuba** | | | | | |
| M±Std | 0.015±0.063 | 0.079±0.124 | -0.041±0.138 | -0.148±0.093 | - |
| 95% CI [L-U] | -0.11 – 0.14 | -0.16 – 0.32 | -0.31 – 0.23 | -0.33 – 0.03 | - |
| P value | **0.012** | 0.526 | 0.768 | 0.11 | - |
| **Nubian** | | | | | |
| M±Std | 0.190±0.063 | 0.254±0.124 | 0.135±0.138 | 0.027±0.093 | 0.175±0.081 |
| 95% CI [L-U] | 0.07 – 0.31 | 0.01 – 0.50 | -0.14 – 0.40 | -0.15 – 0.21 | 0.02 – 0.33 |
| P value | **0.003** | 0.241 | 0.329 | 0.771 | 0.63 |

**Additional file 1:**

**Table S1:** Subgroup analysis of *IL-17A* genotypes distribution across the different Sudanese ethnic groups.

**M±Std**: Mean Difference ± Standard Error. **95% CI [L-U]**: 95% Confidence Interval [ Lower bound -Upper bound].
