# Supplementary material for: Frequency distribution of IL-17A G197A (rs2275913) and IL-17F A7488G (rs763780) polymorphisms among healthy Sudanese population
Source: BMC Res Notes. 2020 Jul 2;13:317. doi: 10.1186/s13104-020-05165-4 (PMC7330939; doi:10.1186/s13104-020-05165-4)
Supplement: Supplementary file 2 — Additional file 2: Table S2. Subgroup analysis of IL-17F genotypes distribution across the different Sudanese ethnic groups. M±Std: Mean Difference ± Standard Error. 95% CI [L-U]: 95% Confidence Interval [ Lower bound -Upper bound]. [file 13104_2020_5165_MOESM2_ESM.docx]

|  | **Arab** | **Beja** | **Fallata** | **Fur** | **Nuba** |
| --- | --- | --- | --- | --- | --- |
| **Beja** | | | | | |
| M±Std | 0.014±0.095 | - | - | - | - |
| 95% CI [L-U] | -0.17 – 0.20 | - | - | - | - |
| P value | 0.886 | - | - | - | - |
| **Fallata** | | | | | |
| M±Std | 0.017±0.107 | 0.004±0.140 | - | - | - |
| 95% CI [L-U] | -0.19 – 0.23 | -0.27 – 0.28 | - | - | - |
| P value | 0.871 | 0.978 | - | - | - |
| **Fur** | | | | | |
| M±Std | -0.036±0.065 | -0.050±0.110 | -0.053±0.121 | - | - |
| 95% CI [L-U] | -0.16 – 0.09 | -0.27 – 0.17 | -0.29 – 0.18 | - | - |
| P value | 0.582 | 0.654 | 0.660 | - | - |
| **Nuba** | | | | | |
| M±Std | -0.028±0.053 | -0.042±0.104 | -0.046±0.115 | -0.008±0.077 | - |
| 95% CI [L-U] | -0.13 – 0.08 | -0.25 – 0.16 | -0.27 – 0.18 | -0.14 – 0.16 | - |
| P value | 0.591 | 0.685 | 0.691 | 0.923 | - |
| **Nubian** | | | | | |
| M±Std | 0.003±0.053 | -0.011±0.104 | -0.015±0.115 | 0.038±0.077 | 0.031±0.067 |
| 95% CI [L-U] | -0.10 – 0.11 | -0.21 – 0.19 | -0.24 – 0.21 | -0.11 – 0.19 | -0.10 – 0.16 |
| P value | 0.962 | 0.915 | 0.897 | 0.620 | 0.646 |

**Additional file 2:**

**Table S2:** Subgroup analysis of *IL-17F* genotypes distribution across the different Sudanese ethnic groups.

**M±Std**: Mean Difference ± Standard Error. **95% CI [L-U]**: 95% Confidence Interval [ Lower bound -Upper bound].
